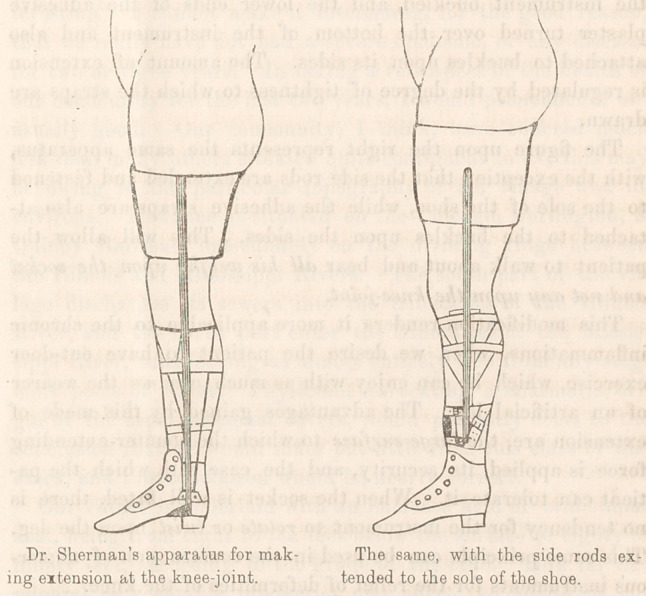# The Mechanical Treatment Necessary in Inflammation of the Knee-joint; with a Description of a New Apparatus for Making Extension

**Published:** 1867-01

**Authors:** Julien S. Sherman

**Affiliations:** Chicago, Ill.


THE
### CHICAGO MEDICAL EXAMINER.

N. S. DAVIS, MD, EDITOR.

VOL. VIII. JANUARY, 1867. NO. 1.

### Original Contributions.

### ARTICLE I.

### THE MECHANICAL TREATMENT NECESSARY IN INFLAMMATION OF THE KNEE-JOINT; WITH A DESCRIPTION OF A NEW APPARATUS FOR MAKING EXTENSION.

BY JULIEN S. SHERMAN, M.D., Chicago, Ill.

The exposed position of this articulation, the thinness of the tissues surrounding, and the amount of labor performed by it, render it a very frequent seat of those diseases and deformities consequent upon injury. Inflammation of this joint is generally more severe and disastrous than of most others, on account of its large size and the extent of the synovial sack involved in the disease. The well-known pathological fact, that inflammation of joints is always followed by reflex contraction of the muscles in its vicinity, is well exemplified in the knee by the strong contraction of the powerful flexors, bending the leg frequently to a right angle with the thigh.

The injurious effect of pressure is also well shown, and the necessity for its removal as urgent as in hip-disease. Specific inflammations are sometimes met with, but are not as frequent as generally supposed, most cases being traumatic in their origin. Yet inflammations occurring in constitutions either scrofulous or syphilitic, are more prone to suppuration and caries than when this element is absent.

Notwithstanding the advantages of medical treatment, there are indications which must be overcome by mechanical means. They are not only necessary for subduing disease in its early developement, but are indispensable for the correction and prevention of deformities following in severe cases. Inflammation of this joint, even in its first stage, is always accompanied by contraction of the flexors, aggravating the pain and increasing the pressure, thereby either wholly preventing, or greatly retarding, spontaneous recovery. This contraction increases with the violence of the inflammation, and should be overcome by tenotomy of all the tendons offering resistance to the extension of the limb. If ether is administered, the particular tendons requiring division should be ascertained before anæsthesia is produced, as, when that stage is reached, the muscles become relaxed, and we may be at loss to determine where division is necessary. Special care must be taken to avoid wounding the peroneal nerve, situated just internal to the biceps tendon. This tendon should be divided from without inwards, at the same time that extension is being made, the sheath and inner fibres of which will then be ruptured before the knife passes completely through, and all danger to the nerve avoided. The limb should then be placed upon an air-cushion, protected by oil-silk, in order that local dressings may be used without soiling the bedding.

*The relief of pressure in inflamed joints is the most important part of the treatment;* the diseased surfaces must be separated to allow of recovery or to prevent unfavorable results. The means for accomplishing this desired end are strictly mechanical. Adhesive straps may be applied to the leg, below the knee, and the surfaces of the joints separated by making extension with the pulley and weight, as in adhesive strap dressing for fractures of the thigh. The limb may be placed in the horizontal position or upon an inclined plane, as circumstances may indicate. This treatment will be found to greatly relieve the pain and entirely remove the pressure. If it be adopted at the outset of the attack, the flexing of the limb will be prevented and division of the tendons rendered unnecessary. Should suppuration occur and the joint become greatly distended with pus, it should be carefully evacuated by means of a trochar, avoiding the admission of air into the synovial sack, and the extension persevered in. Moderate pressure upon the femoral artery is advised by good authority, as diminishing the flow of blood to the part, but it is difficult to maintain and frequently adds to the discomfort of the patient.

Dr. Sherman's apparatus for making extension at the knee-joint.

The same, with the side rods extended to the sole of the shoe.

The accompanying cuts represent an apparatus for making extension. It consists of a *wooden socket*, constructed to accurately fit the thigh and similar to those used for artificial legs, against this the counter-extension is made, and thus evenly distributed over the thigh and tuberosity of the ischium. A steel rod is attached to each side of the socket, reaching to within a few inches of the ankle, and the two rods are joined behind by a broad band of sheet-iron, which is moulded to fit the posterior part of the leg; on the front, and joining the sheet-iron band, is a strap which, being buckled, holds the leg firmly in the apparatus. It is applied as follows:—Six adhesive straps are cut, two inches in width at the top and tapering to one at the bottom, and should be long enough to reach from about one inch below the knee to the ankle; they are then applied to the leg, as represented in the cut, and secured by a bandage; the socket is then placed upon the thigh, the strap at the bottom of the instrument buckled, and the lower ends of the adhesive plaster turned over the bottom of the instrument and also attached to buckles upon its sides. The amount of extension is regulated by the degree of tightness to which the straps are drawn.

The figure upon the right represents the same apparatus, with the exception that the side rods are extended and fastened to the sole of the shoe, while the adhesive straps are also attached to the buckles upon the sides. This will allow the patient to walk about and bear *all his weight upon the socket and not any upon the knee-joint.*

This modification renders it more applicable to the chronic inflammations, when we desire the patient to have out-door exercise, which he can enjoy with as much ease as the wearer of an artificial leg. The advantages gained by this mode of extension are, the *large surface* to which the counter-extending force is applied, its security, and the ease with which the patient can tolerate it. When the socket is well fitted, there is no tendency for the instrument to *rotate* or *twist* upon the leg. This same principle can be used in the construction of numerous instruments for the relief of deformities of the knee.

## Figures and Tables

**Figure f1:**